# Changes in Ecophysiology, Osmolytes, and Secondary Metabolites of the Medicinal Plants of *Mentha piperita* and *Catharanthus roseus* Subjected to Drought and Heat Stress

**DOI:** 10.3390/biom10010043

**Published:** 2019-12-27

**Authors:** Haifa A. Alhaithloul, Mona H. Soliman, Keshav Lalit Ameta, Mohamed A. El-Esawi, Amr Elkelish

**Affiliations:** 1 Biology Department, College of Science, Jouf University, Sakaka 2014, Saudi Arabia; Haifasakat2030@gmail.com; 2 Botany and Microbiology Department, Faculty of Science, Cairo University, Giza 12613, Egypt; monahsh1@gmail.com; 3 Department of Chemistry, School of Liberal Arts and Sciences, Mody University of Science and Technology, Lakshmangarh 332311, Rajasthan, India; klameta77@hotmail.com; 4 Botany Department, Faculty of Science, Tanta University, Tanta 31527, Egypt; mohamed.elesawi@science.tanta.edu.eg; 5 Botany Department, Faculty of Science, Suez Canal University, Ismailia 41522, Egypt

**Keywords:** secondary metabolites, drought, heat stress, *Mentha piperita*, *Catharanthus roseus*

## Abstract

Global warming contributes to higher temperatures and reduces rainfall for most areas worldwide. The concurrent incidence of extreme temperature and water shortage lead to temperature stress damage in plants. Seeking to imitate a more natural field situation and to figure out responses of specific stresses with regard to their combination, we investigated physiological, biochemical, and metabolomic variations following drought and heat stress imposition (alone and combined) and recovery, using *Mentha piperita* and *Catharanthus roseus* plants. Plants were exposed to drought and/or heat stress (35 °C) for seven and fourteen days. Plant height and weight (both fresh and dry weight) were significantly decreased by stress, and the effects more pronounced with a combined heat and drought treatment. Drought and/or heat stress triggered the accumulation of osmolytes (proline, sugars, glycine betaine, and sugar alcohols including inositol and mannitol), with maximum accumulation in response to the combined stress. Total phenol, flavonoid, and saponin contents decreased in response to drought and/or heat stress at seven and fourteen days; however, levels of other secondary metabolites, including tannins, terpenoids, and alkaloids, increased under stress in both plants, with maximal accumulation under the combined heat/drought stress. Extracts from leaves of both species significantly inhibited the growth of pathogenic fungi and bacteria, as well as two human cancer cell lines. Drought and heat stress significantly reduced the antimicrobial and anticancer activities of plants. The increased accumulation of secondary metabolites observed in response to drought and/or heat stress suggests that imposition of abiotic stress may be a strategy for increasing the content of the therapeutic secondary metabolites associated with these plants.

## 1. Introduction

One of the biggest issues concerning the impact of climate change on human being and natural ecosystems is the reaction of the Earth’s hydrologic cycle to global warming [1,2]. As sessile species, plants are exposed to unavoidable drought and heat stress, which leads to growth and yield reductions in important crop plants [3]. The ongoing effects of climate change are expected to increase the frequency of drought and heat extremes, and the combined effects of these stresses are predicted to be more damaging than their individual effects [3]. Plants experience drought either due to low rainfall or less availability of water in the soil [4], and the intensity of drought is exacerbated by increases in temperature. Plants can endure periods of low water availability and high temperature by two strategies: avoidance and tolerance [5,6]. Some tolerance mechanisms are specific to a particular stress, while others are commonly initiated in response to variety of stresses. Examples of the latter mechanism are the accumulations of different metabolites, especially amino acids and sugars [7,8].

Stress mitigation responses are complex processes involving several factors, including sensing and signaling mediated by transcription factors, modulation of hormonal levels, and production of secondary metabolites [7,9]. Secondary metabolites are of particular interest, as they have important functions in regulating plant environmental interactions and subsequent adaptation responses [10,11,12]. Enhanced synthesis of phenylpropanoid metabolites like scopolin, sinapic acid, sinapoyl aldehyde, and flavonoids contribute to the synthesis of important structural compounds like lignins, but they also reduce oxidative damage and prevent photoinhibition by contributing to the antioxidant potential under abiotic stresses like drought and heat [13,14].

Plant secondary metabolites also exhibit remarkable biological activities in non-plant species and are used as medicines and food additives due to their aromatic, therapeutic, and culinary properties. However, their synthesis and accumulation is complicated and is influenced by many different genetic, morphogenic, and environmental factors [12,15,16,17]. For this reason, the observation that abiotic stress induces changes in the synthesis of secondary metabolites suggests that the imposition of a stress may be a strategy for enhancing the therapeutic properties of many medicinal plant species [18,19]. However, the success of this strategy would depend on establishing a balance between the synthesis of the medicinal substances of interest and the synthesis of the other stress metabolites needed by the plant to tolerate the stress [20,21]. Ecophysiological studies must therefore also be part of this strategy, but the ecophysiology of stress responses in medicinal plants has received little attention [22].

Two important medicinal plants are peppermint (*Mentha piperita*) and Madagascar periwinkle (*Catharanthus roseus*). *Mentha piperita*, a popular herb in the Lamiaceae family, is cultivated for its flavoring and fragrance properties and is used in the production of food, cosmeceuticals, personal hygiene products, and other pharmaceutical products [23,24]. Peppermint oil has therapeutic properties and is used in aromatherapy, mouthwashes, and toothpastes. It is also an active ingredient in several topical preparations used to calm pruritus and relieve irritation and inflammation [23]. Key components of peppermint oil are menthol and menthone, with lesser concentrations of pulegone, menthofuran, and limone [24]. *Catharanthus roseus* is an important medicinal and ornamental plant from the Apocynaceae and is a key source of the anticancer drugs vincristine and vinblastine, as well as nearly seventy other alkaloids [25]. However, the responses of these two medicinal plants to abiotic stresses are unknown.

The aims of the present study were therefore to document the ecophysiological responses of *M. piperita* and *C. roseus* to drought and/or heat stress and to assess any stress-related changes in osmolytes and secondary metabolites. A secondary goal was to confirm the medicinal activities of aqueous and methanolic extracts from these plants against pathogenic bacteria, fungi, and cancer cells. The findings indicate that exposure of *C. roseus* and *M. piperita* to abiotic stress (heat and/or drought) redirects plant metabolism in a manner that could potentially increase the synthesis of the therapeutic secondary metabolites that could have antimicrobial and in vitro anticancer activity.

## 2. Materials and Methods

### 2.1. Pot Experiments

Sterilized seeds of *Mentha piperita* (peppermint) and *Catharanthus roseus* (Cape periwinkle) were sown in plastic pots (19 cm in diameter × 17 cm in depth) filled with a 5:1 (*v*/*v*) mixture of clay soil and peat. After germination, the seedlings were well-watered until the age of 45 (*M. piperita)* or 60 (*C. roseus*) days. Thereafter, plants were subjected to heat and/or drought stress for seven and fourteen days. Pots were kept in a greenhouse maintained at 65% humidity and a 12 h photoperiod. Therefore, we had following set of treatments:

Control: Plants grown under normal (17–22 °C) temperature and normal irrigation.

Heat stress (H): Plants grown at high temperature (35 °C).

Drought stress (D): 50% drought stress imposed by the cessation of watering for 2 weeks.

Drought and heat stress (H+D): This combined stress occurred at the same time and imposed by transferring the plants to high temperature (35 °C) as well as cessation of watering for 2 weeks.

### 2.2. Seedling Growth Measurements

Morphological traits of treated and untreated of *M. piperita* and *C. roseus* plants were measured. Five plants with roots were harvested and transferred to the laboratory for measuring plant height; shoot and root fresh weight. Shoot and root dry weights were measured after drying in the shade at room temperature for 72 h.

### 2.3. Biochemical Assays

#### 2.3.1. Total Soluble Proteins

Fresh leaves (0.5 g) of *M. piperita* and *C. roseus* harvested at 7 and 14 days from all treatments were ground in 1 mL of phosphate buffer (0.1 M, pH 7.0) and kept in ice. The protein concentration was determined following Bradford assay [26]. Absorbance was read at 595 nm on a spectrophotometer.

#### 2.3.2. Proline Content

Proline was measured according to Troll and Lindsley [27] with slight modification. Samples (100 mg fresh weight) of *M. piperita* and *C. roseus* leaves were harvested at 7 and 14 days, ground in liquid nitrogen, homogenized in 5% (*w*/*v*) sulfosalicylic acid and centrifuged at 14,000× *g* for 5 min. The supernatants were mixed with 2 mL 40% methanol, and heated at 95 °C in water bath for 60 min. After cooling, 1 mL of extract was added to 1 mL of acetic acid and 1 mL of a mixture containing 120 mL distilled water and 300 mL phosphoric acid. The samples were boiled for 30 min, cooled, and then 5 mL of toluene was added. The upper phase was recovered, and the absorbance was measured at 528 nm. The proline concentration was estimated from a standard curve.

#### 2.3.3. Glycine Betaine

A 0.5 g sample of *M. piperita* or *C. roseus* leaves harvested at 7 and 14 days was homogenized with 10 mL of Milli-Q Water and was incubated for 24 h at 25 °C. The homogenate was filtered, and the filtrate was mixed with 2 N sulfuric acid at a ratio of 1:1 (*v*:*v*). This mixture (0.5 mL) was placed on ice for 1 h and then 0.2 mL of cold potassium tri-iodide reagent was added. After storage at 4 °C for 16 h, the mixture was centrifuged at 14,000 rpm for 15 min at 0 °C. Absorbance of the supernatant was read at 365 nm. The glycine betaine concentration was estimated from a standard curve [28].

### 2.4. Phytochemical Assays

#### 2.4.1. Total Soluble Sugars

Total soluble sugars (TSS) were extracted from *M. piperita* and *C. roseus* leaves at 7 and 14 days for all stress treatments using the method of Irigoyen et al. [29]. A sample (0.2 g) of dried leaves was homogenized in 5 mL of 96% (*v*/*v*) ethanol and washed with 5 mL of 70% (*v*/*v*) ethanol. The extract was centrifuged at 3500× *g* for 10 min, and the supernatant was stored at 4 °C prior to measurement. Each TSS concentration was determined by reacting 0.1 mL of the ethanolic extract with 3 mL of freshly prepared anthrone reagent (150 mg anthrone plus 100 mL of 72% sulfuric acid) by placing it in a boiling water bath for 10 min. After cooling, the mixture absorbance was recorded at 625 nm.

#### 2.4.2. Mannitol and Inositol Contents

The mannitol content in stressed and unstressed *M. piperita* and *C. roseus* plant leaves was determined using a Mannitol Colorimetric Assay kit (Sigma-Aldrich, Munich, Germany) and measuring the absorbance at 450 nm. The inositol content in control and stressed *M. piperita* and *C. roseus* leaves were measured with a Myo-Inositol Assay kit (BioVision, Inc., San Francisco, CA, USA).

#### 2.4.3. Total Phenolic Content (TPC)

The total phenolic content (TPC) was estimated in fresh samples of the uppermost leaves in *M. piperita* and *C. roseus* following the protocol of Slinkard and Singleton [30]. After extraction with ethanol (80%, *v*/*v*), the supernatant was reacted with Folin and Ciocalteau’s reagent [31] and the optical density of the mixture was read at 750 nm. The concentrations were estimated from a standard curve of pyrogallol.

#### 2.4.4. Total Flavonoid Content (TFC)

The total flavonoid content (TFC) was estimated according to Zhishen et al. [32] using catechin as a standard. Samples were extracted in methanol and the absorbance was recorded at 510 nm. The flavonoid content was expressed as mg per g fresh weight (FW).

#### 2.4.5. Saponins

Saponins were determined according to the method of Obadoni and Ochuko [33]. A 10 g sample of air-dried *M. piperita* or *C. roseus* leaf material was put into a conical flask containing 50 mL of 20% aqueous ethanol. The samples were heated over a hot water bath at about 55 °C for 4 h with continuous stirring. The mixture was filtered, and the residue was re-extracted with another 100 mL of 20% ethanol. The combined extracts were reduced to 20 mL over a water bath at about 90 °C. The concentrate was transferred into a 250 mL separating funnel and 10 mL of diethyl ether was added and shaken vigorously. The aqueous layer was recovered, and the ether layer was discarded. The purification process was repeated, and then 30 mL of n-butanol was added. The combined n-butanol extracts were washed twice with 10 mL of 5% aqueous sodium chloride and heated in a water bath. After evaporation, the samples were dried in an oven to a constant weight and the saponin content was calculated as percentage.

#### 2.4.6. Terpenoids

Total terpenoids were estimated by the protocol of Ferguson [34]. A 10 g sample of air-dried *M. piperita* or *C. roseus* leaf material was soaked in alcohol for 24 h and then filtered. The filtrate was extracted with petroleum ether, and the ether extract was treated as the total terpenoid extract.

#### 2.4.7. Tannins

Tannins were determined as described previously [35,36]. A 1 g sample of powdered air-dried leaf from *M. piperita* and *C. roseus* was placed in a conical flask can combined with 100 mL of distilled water. This was boiled gently for 1 h on an electric hot plate and then filtered through Whatman 42 filter paper (125 mm) into a 100 mL volumetric flask. A 5.0 mL volume of Folin-Denis reagent and 10 mL of saturated Na_2_CO_3_ solution were added to 50 mL of distilled water in a 100 mL conical flask, and 10 mL of diluted extract was added for color development. The solution was left to react for 30 min in a water bath at 25 °C after thorough agitation. Optical density was read at 700 nm and compared against a standard curve of tannic acid:

$$Tannic\;acid\;\left(\mathrm{mg}/100\;g\right)=\;\frac{C\;\times \;extract\;volume\;\times \;100}{Aliquot\;volume\;\times \;weight\;of\;sample}$$

where *C* is the concentration of tannic acid read off the graph.

#### 2.4.8. Alkaloids

Alkaloids were determined according to Harborne [37]. A 5 g sample of air-dried *M. piperita* or *C. roseus* leaf was weighed into a 250 mL beaker, 200 mL of 10% acetic acid in ethanol was added, and the beaker was covered and allowed to stand for 4 h. The solution was filtered, and the extract was concentrated on a water bath to one-quarter of the original volume. Concentrated ammonium hydroxide was added dropwise to the extract until precipitation was complete. The whole solution was allowed to settle, and the precipitate was collected and washed with dilute ammonium hydroxide and then filtered. The residue, which was designated as the total alkaloids, was dried and weighed.

### 2.5. Antioxidant Assays

#### 2.5.1. Preparation of the Aqueous and Methanolic Extracts

A 10 g sample of air-dried aerial parts (dried for one week at room temperature) from healthy *M. piperita* or *C. roseus* plants was ground to a fine powder. A 5 g sample of the powder was then extracted overnight in 10 mL of distilled water or pure methanol. The extracts were filtered through Whatman No. 1 filter paper and stored at 4 °C.

#### 2.5.2. DPPH Scavenging Activities of *M. piperita* and *C. roseus* Extracts

Total antioxidant capacity was measured by determining the 2,2-diphenyl-1-picrylhydrazyl (DPPH) scavenging activity of aqueous and methanolic extracts (50 mg/mL) of *M. piperita* and *C. roseus* according to Espin et al. [38] with slight modifications. Dried plant extract was diluted in pure methanol at different concentrations, and 2 mL were added to 0.5 mL of a 0.2 mM DPPH methanolic solution. The mixture was shaken vigorously and left to stand at room temperature for 30 min. The absorbance of the resulting solution was then measured at 517 nm measured after 30 min.

$$Antioxidant\;activity\;\%=\;\frac{\left(Absorbance\;Control-Absorbance\;Sample\right)\;\times \;100}{Absorbance\;Control}$$

### 2.6. Determination of Antimicrobial Activity

#### 2.6.1. Antibacterial Activities of *M. piperita* and *C. roseus* Extracts

The antibacterial activity of different concentrations (10% and 20%) of the aqueous and methanolic extracts of *M. piperita* and *C. roseus* were evaluated against *Pseudomonas aeruginosa, Staphylococcus aureus* and *Ralstonia solanacearum* using the agar well diffusion method. Autoclaved agar plates were inoculated with bacterial cell suspensions and wells (5 mm) were made in each agar plate using a sterile metallic borer. Each well was loaded with 25 µL of extract or ampicillin (0.2 µg/mL) as positive control. All the plates were incubated at 37 °C for 48 h, and the results were expressed as the size of the zone of inhibition.

#### 2.6.2. Antifungal Activities of *M. piperita* and *C. roseus* Extracts

The antifungal activity of different concentrations (0.5% and 1.0%; *v*/*v*) of *M. piperita* and *C. roseus* aqueous and methanolic extracts were investigated against the plant pathogenic fungi *Fusarium oxysporum* and *Aspergillus terreus* by the agar well diffusion method. The chemical fungicide Rhizolex-T (20 ppm) was used as a positive control. Agar medium was poured into 90-mm Petri dishes and inoculated with a 5-mm disk of *Fusarium oxysporum* and *Aspergillus* culture. Five replicates (dishes) were used for each treatment concentration and control, and the dishes were incubated at 25 °C for 5–7 days. The reduction in mycelial growth was calculated as percentage of the control fungal growth.

### 2.7. Cytotoxic and Anticancer Activities of M. piperita and C. roseus Extracts

Cytotoxicity against the PC3 human prostate cancer and the MCF-7 human breast cancer lines was measured in vitro using the 3-(4,5-dimethylthiazol-2-yl)-2,5-diphenyltetrazolium bromide (MTT) assay, as described by Romijn et al. [39]. Cells were exposed to different concentrations (50, and 100 µg/mL) of *M. piperita* and *C. roseus* aqueous and methanolic extracts by plating in triplicate in 96-well plates in serum-free media containing either plant extract or 5-fluorouracil (20 µg/mL; positive control). After incubation at 37 °C for 72 h, 20 µL of MTT solution was added, the cells were incubated for 3 h, and the absorbance was read at 570 nm in a multi-mode plate reader (BioTek, Bad Friedrichshall, Heilbronn, Germany). The anticancer activity of the extract was calculated using the following formula:

$$Inhibition\;\%=\frac{100-optical\;density\;of\;sample\;\times \;100}{Optical\;density\;of\;control}$$

### 2.8. Statistical Analysis

Data are the means ± standard error (SE). Analysis of variance (ANOVA) was carried out using SPSS version 19 (SPSS Inc., Chicago, IL, USA). Tukey’s test was conducted to test the significance between mean values (*p* < 0.05).

## 3. Results and Discussion

### 3.1. Effects of Drought and Heat Stress on the Growth and Physiological and Metabolomic Traits of M. piperita and C. roseus

Drought and heat stress frequently co-occur, especially in arid and semi-arid regions of globe, and they result in significant decreases in plant growth and productivity [40,41]. Plants frequently survive these stress conditions by changing their metabolism to favor the synthesis of osmolytes and secondary metabolites that promote stress tolerance. Previous studies on drought [42] and heat stress [43,44] in crop plants like wheat and *Lens culinaris* have demonstrated significant reductions in growth and development due to damage to cell membranes and disruption of photosynthesis and water relations. However, the ecophysiological and metabolic responses of important medicinal plants to abiotic stress have received little attention. The present study was prompted by this gap in knowledge.

The present findings confirm that both *M. piperita* and *C. roseus* show typical growth responses to abiotic stress, including decreases in plant height and in fresh and dry weight, and that the effects are most pronounced in seedlings exposed to a combined heat and drought stress (Table 1 and Table 2). The maximum declines in height and fresh and dry weight in the shoot were 28.99, 44.88, and 45.45% in *M. piperita* and 48.63, 33.68, and 42.58% in *C. roseus* following exposure to a combined heat and drought stress. No significant differences were observed in the root fresh and dry weight in the stressed plants when compared to control plants (Table 1 and Table 2).

Drought and heat stress restrict the uptake and assimilation of nutrients, while favoring amino acid accumulation, resulting in significant effects on plant growth and development [45,46]. High temperatures also alter cell growth and proliferation by influencing the cell cycle progression and hence cell division [47]. Growth inhibition triggered by extreme environmental conditions has been associated with up-regulation and down-regulation of cell cycle inhibitors and stimulators [48]. Plants are able to tolerate abiotic stress and to prevent some of the stress-mediated retardation of growth and development by accumulating specific organic and inorganic osmolytes, including proline, glycine betaine, sugars, and sugar alcohols like inositol and mannitol [5].

**Figure 1:**
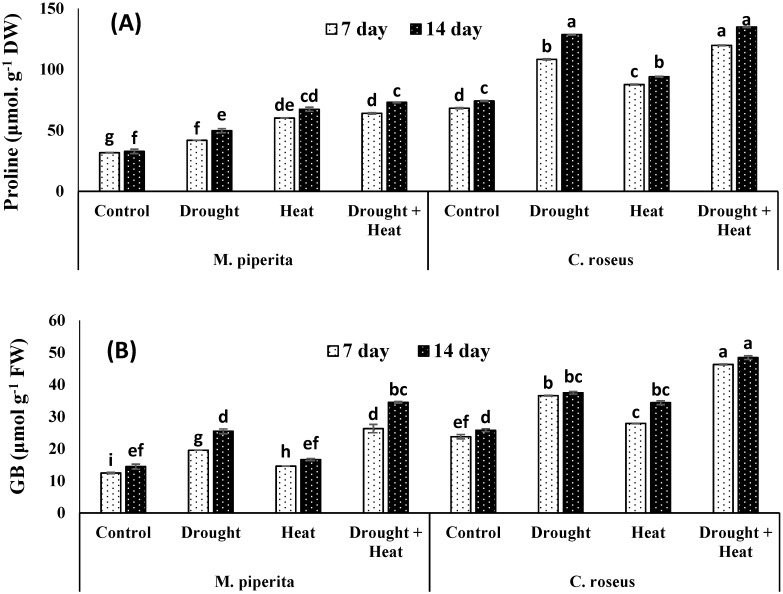
Effect of drought, heat and drought + heat stress on the proline (**A**) and glycine betaine (**B**) of *M. piperita* and *C. roseus* after seven and fourteen days of stress exposure. Mean (±SE) of five values is presented and values denoted by different letters are significantly different at *p* < 0.05.

**Figure 2:**
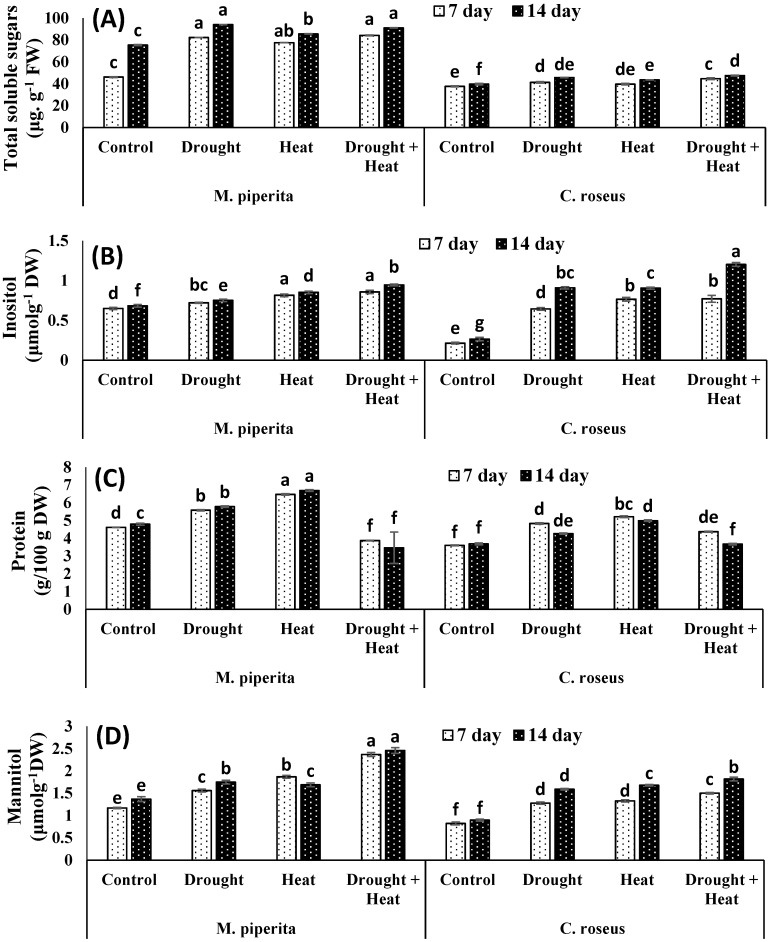
Effect of drought, heat and drought + heat stress on the total soluble sugars (**A**), inositol (**B**), protein (**C**) and mannitol (**D**) of *M. piperita* and *C. roseus* after seven and fourteen days of stress exposure. Mean (±SE) of five values is presented and values denoted by different letters are significantly different at *p* < 0.05.

In present study, both *M. piperita* and *C. roseus* accumulated significant amounts of proline, glycine betaine, sugar, inositol, and mannitol in response to drought and heat stress, with maximal accumulations observed in response to the combined drought and heat stress (Figure 1 and Figure 2). The respective increases in proline, glycine betaine, sugar, inositol, and mannitol over the control values were 34.13, 43.22, 19.89, 9.04 and 21.71% in *M. piperita* and 42.34, 31.17, 12.93, 70.87 and 43.45% in *C. roseus* after 14 days of drought stress. Protein contents were increased maximally in response to the individual drought or heat stress exposures. These results could be attributed to the induction of genes involved in protein synthesis and heat shock proteins by the individual drought or heat stress. In contrast, the combined drought and heat stress reduced protein content which might be due to the increase in severity which in turn led to decreasing or inhibiting protein biosynthesis as well as down-regulation of heat shock proteins. Accumulation of all these osmolytes was maximal after fourteen days of stress exposure. Similar accumulations of osmolytes were also observed after 14 days of heat stress. The respective maximal accumulations were 55.09, 58.09, 17.25, 27.69 and 44.30% in *M. piperita* and 45.04, 46.77, 16.42, 77.97 and 50.68% in *C. roseus* following the combined heat and drought stress (Figure 2).

Osmolytes help plants to avert the damaging effects of drought and heat stress by increasing the cellular water content to maintain the structural and functional integrity of cells [9,49]. The accumulation of osmolytes, even at high concentrations, does not affect cell function because of the compatible nature of these solutes [50,51]. Prominent osmolytes include a variety of amino acids, sulfonium and ammonium compounds, sugars, and polyhydric alcohols. Accumulation of compatible osmolytes prevents osmotic shock, protects enzyme activity, and ameliorates oxidative damage by scavenging stress-induced reactive oxygen species [49].

Proline accumulation alleviates cytoplasmic acidosis and maintains the NADP+/NAD+ ratio [52]. Excess proline also serves as a sink for excess reductants and assists in photosynthesis and respiration by increasing the availability of NADP and NAD. Increases in glycine betaine or proline can also prevent photoinhibition by maintaining the carboxylase activity of Rubisco [53,54]. Increases in NADP maintain the operation of the pentose phosphate pathway, leading to a greater generation of NADPH and a supply of the ribose-5-phosphate substrate required for purine synthesis [55,56]. Increased accumulation of proline and sugars under drought stress has been reported in maize [57] and wheat [8], and combined exposure to drought and heat stress increased the accumulation of sugars in *Lens culinaris* [46]. Increased accumulation of osmolytes is a direct result of the up-regulation of their biosynthesis and down-regulation of their catabolic pathways, as reported for proline metabolism in *Brassica juncea* [58]. In *Vigna aconitifolia,* Harsh et al. [59] have reported that the increased accumulation of proline and sugars in response to heat stress reflects a greater antioxidant potential. In *Cicer arietinum* L., increased accumulation of proline during heat stress has been reported to protect the functioning of major enzymes like Rubisco and enzymes involved in sugar metabolism, including sucrose phosphate synthase and invertase [60]. Plants that accumulate proline, trehalose, fructans, mannitol, or glycine betaine exhibit improved stress tolerance [52,61,62,63].

Sugars display hormone-like activities and can function as primary messengers in signaling. Sugar-mediated signaling controls growth and developmental events; consequently, high concentrations of accumulated sugars may reflect undesirable growth conditions during early seedling development [64], whereas their accumulation at later growth stages has been reported to improve cellular and whole plant functioning under both normal and stress conditions [42]. Increased accumulation of sugars and other osmolytes contributes to growth regulation under stress conditions by elimination of reactive oxygen species (ROS) through a strengthened antioxidant system [65,66]. In the present study, soluble sugars were enhanced under drought and heat stress, which might be attributed to the enhancement of glucose and fructose as a result of increasing the hydrolysis of the sucrose and starch. Soluble sugars might do other numerous cellular and nutritional functions as an energy source and osmoprotectants which protect the plants under stress conditions.

**Figure 3:**
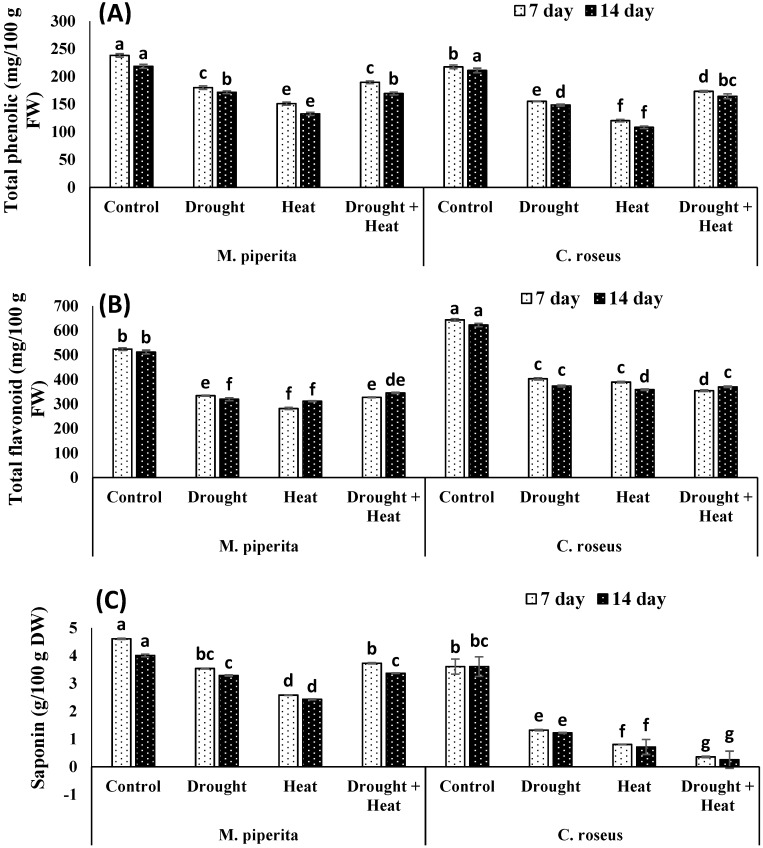
Effect of drought, heat and drought + heat stress on the total phenols (**A**), total flavonoids (**B**) and saponins (**C**) in *M. piperita* and *C. roseus* after seven and fourteen days of stress exposure. Mean (±SE) of five values is presented and values denoted by different letters are significantly different at *p* < 0.05.

**Figure 4:**
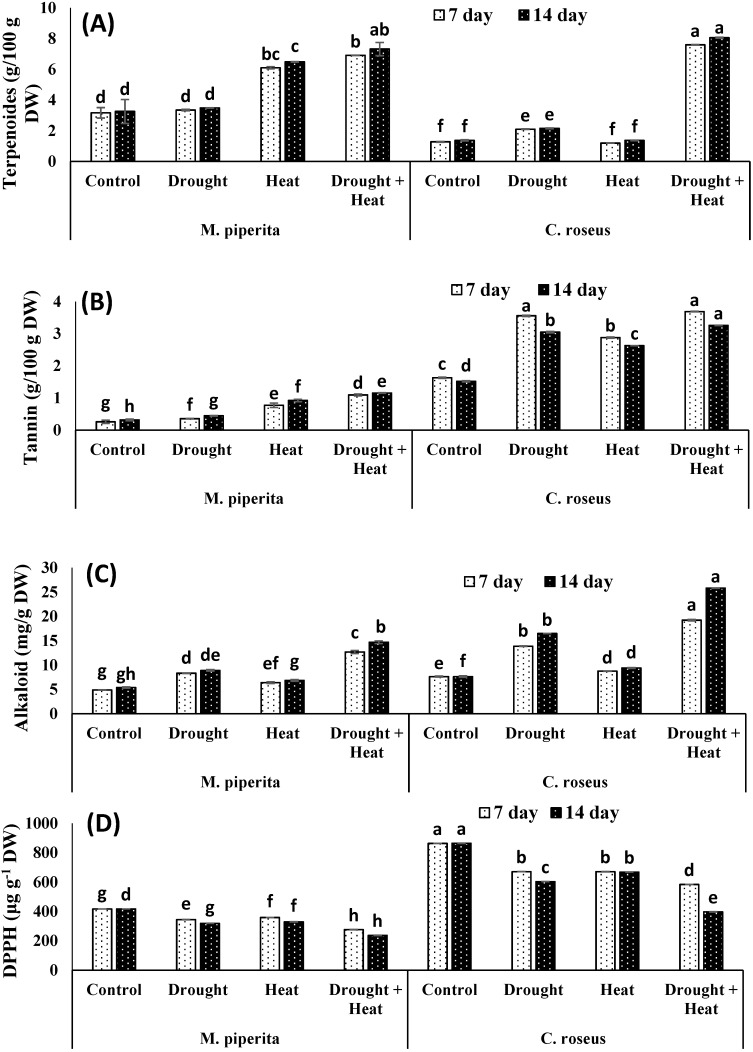
Effect of drought, heat and drought + heat stress on the terpenoids (**A**), tannins (**B**), alkaloids (**C**) and DPPH scavenging activities (**D**) in *M. piperita* and *C. roseus* after seven and fourteen days of stress exposure. Mean (±SE) of five values is presented and values denoted by different letters are significantly different at *p* < 0.05.

Drought and heat stress had differential effects on the accumulation of secondary metabolites. Total phenols, flavonoids, saponins and DPPH scavenging activities underwent significant declines in response to drought and heat stress, whereas terpenoid, alkaloid, and tannin levels increased, with the greatest increase seen in plants exposed to the combined stress (Figure 3 and Figure 4). After fourteen days of the combined stress, total phenols, flavonoids, and saponins showed respective decreases of 22.51, 32.55, and 15.96% in *M. piperita* and 22.13, 40.51, and 92.99% in *C. roseus*. The maximal declines of 39.29% and 48.67% for total phenols and 39.14% and 42.37% for flavonoids in *M. piperita* and *C. roseus,* respectively, were observed in heat-stressed plants after fourteen days of stress exposure (Figure 3 and Figure 4). The drought-stressed *M. piperita* and *C. roseus* plants showed respective declines of 21.46% and 29.57% in total phenols, 37.57% and 39.96% in flavonoids, and 17.95% and 66.20% in saponins (Figure 3 and Figure 4), whereas tannins, alkaloids, and terpenoids increased in both species due to drought stress. The maximal increases were 29.14% and 50.16% for tannins, 39.39% and 53.72% for alkaloids, and 6.59% and 36.11% terpenoids in *M. piperita* and *C. roseus*, respectively, following exposure to the combined stress.

At both developmental stages (i.e., at seven and fourteen days), secondary metabolite accumulations increased. Secondary metabolites include an array of useful natural products that form important components of plant defense systems to counteract the deleterious effects of environmental stresses [12,67,68]. These metabolites have remarkable biological activities and are often exploited as medicinal and food ingredients for therapeutic, aromatic, and culinary purposes [68]. Increased accumulation of secondary metabolites prevents the oxidative effects of drought stress on the membrane structure and imparts greater pharmacological properties to plants [69]. Drought stress influences the accumulation of key secondary metabolites, including phenols and flavonoids, in *Amaranthus* [70]. In contrast to our results, Gharibi et al. [69] recently demonstrated increased accumulation of flavonoids and expression of flavonoid biosynthesis genes in *Achillea pachycephala* after prolonged exposure to drought; the overall effect enhanced oxidative stress due to the increased accumulation of ROS like H_2_O_2_. Under stress conditions, the accumulation of secondary metabolites helps plants to modulate their response mechanisms as these substances act as key signaling components [71,72]. Increased accumulation of metabolites under stress conditions protects the structural and functional aspects of membranes and cells [72].

In contrast to our results, Liu et al. [73] demonstrated an increased accumulation of alkaloids and the expression of genes regulating secondary metabolite accumulation in drought-stressed *C. roseus*. However, reports discussing the combined effects of drought and heat stress on the accumulation of secondary metabolites are rare. Accumulation of secondary metabolites, including isoprenoids, alkaloids, and phenolics, under stressful conditions occurs due to the oversupply of NADPH + H+ [72,74,75]. In the present study, both plant species exhibited significant accumulations of alkaloids, which presumably play similar roles in drought and heat tolerance as they do in other plants. Plant terpenoids function in a variety of growth and development processes under normal and stress environments [76]. Genes regulating the shikimic acid pathway and the biosynthesis of alkaloids like anthocyanins and lignin are up-regulated in response to stress [77]. In *Betula pendula* and *Populus tremula*, Ibrahim et al. [78] demonstrated increased accumulation of terpenoids at elevated temperatures. In *Pinus radiata*, Escandón et al. [79] demonstrated that the accumulation of secondary metabolites, including flavonoids and terpenoids, is regulated by zeatin riboside and isopentenyl adenosine. The *piperita* and *C. roseus* growing under control conditions shown to be more efficient in scavenging DPPH free radicals and had a higher reducing power than those exposed to drought and heat stress. This finding provides evidence that tissues of *piperita* and *C. roseus* subjected to heat and/or drought stress contain less antioxidants and reducing compounds. Król and his co-author [80] and Zainol et al. [81] proved that the phenolic compounds are the main contributors to the scavenging activities. The degree of antioxidative impact generated by phenols mostly relies on the structure of provided compound and particularly on the amount and distribution of hydroxyl groups (-OH). Antioxidative activity is substantially greater if a compound has two -OH groups in the ortho position [82].

### 3.2. Effects of Drought and Heat Stress on the Antibacterial, Antifungal, and Anticancer Activities of the Extracts of M. piperita and C. roseus

Both plant species were tested for their medicinal efficacy against certain potentially pathogenic fungal and bacterial species and cancer cell lines. Both aqueous and methanolic extracts of *M. piperita* and *C. roseus* significantly inhibited the growth of bacterial and fungal species, and they both substantially suppressed the growth of cancerous cell lines. The effects on growth of *P. aeruginosa*, *S. aureus* and *R. solanacearum* were most apparent at higher extract concentrations (20%), with the effect much more obvious against *S. aureus.* When compared to *M. piperita* extracts, the *C. roseus* extracts were more effective at inhibiting bacterial growth (Table 3). The aqueous and methanolic extracts of *M. piperita* and *C. roseus* also significantly inhibited the growth of fungal species like *A. terreus*. Again, methanolic extracts were more effective than the aqueous extracts, and the inhibitory effect was concentration dependent, increasing with higher concentrations (Table 4). The results also showed that drought and heat stress significantly reduced the growth inhibition of bacterial and fungal species, and thus reduced the antimicrobial activities and quality of plant species (Table 3 and Table 4).

The extracts of both plant species suppressed the growth of cancerous cells and again showed a concentration dependence with both the aqueous and the methanolic extracts. The inhibitory effect was more much evident in the PC3 cell line, and the *C. roseus* extracts were more effective than the *M. piperita* extracts. The methanol extracts were also more effective than the aqueous extracts at suppressing cancer cell growth (Table 5). The results also showed that drought and heat stress significantly decreased cancer inhibition, and thus reduced the anticancer activities and quality of plants (Table 5).

Singh et al. [83] have also demonstrated a significant inhibitory effect of peppermint extract on the growth of certain gram positive and gram-negative bacteria. The antibacterial and antifungal activities of *M. piperita* are ascribed to presence of the essential oil and other key phytochemical components. Traditionally, peppermint and its oil have been used medicinally for their antispasmodic, antiseptic, and aromatic properties and in the treatment of colds, nausea, cancers, cramps, indigestion, sore throat, and toothache [83,84]. Peppermint oil exhibits *in vitro* antibacterial activity, and various commercial preparations show beneficial activities [85]. Peppermint also exhibits antiviral and fungicidal activities [86]. Peppermint has been reported to reduce the incidence and multiplicity of lung carcinogenicity and mutagenicity [87].

The polyphenolic compounds present in plants are considered to impart their medicinal properties. Among the key polyphenolic compounds with biologically active roles are the hydroxycinnamic acids (HCAs), such as caffeic, p-coumaric, ferulic, and rosmarinic acids. These compounds account for one-third of the total polyphenolics in plants and are responsible for major functions [88]. The medical and other related pharmaceutical functions of these active compounds are ascribed to their protective activity of preventing oxidative damage due to excess ROS [89]. Recently, Alexa et al. [90] have demonstrated a considerable inhibition of the MDA-MB-231 breast carcinoma cell line and the A375 human melanoma cell line, as well as antimicrobial activity against *S. aureus,* due to presence of certain key HCAs in *Mentha piperita* L. and *Lavandula angustifolia*. In present study, both *M. piperita* and *C. roseus* species showed significant presence of phytochemicals including phenols, flavonoids, tannins, and alkaloids.

The current results indicated that *C. roseus* provides antibacterial activity. The extracts displayed antibacterial activity by exhibiting zones of inhibitions versus the pathogenic microorganisms utilized in the study. Plants are a vital resource of several key compounds of medicinal and pharmaceutical importance, and plant-derived medicines have a far higher molecular diversity than synthetic drugs [91]. The presence of alkaloids in *C. roseus* reflects its medicinal activity, including anticancer activity [92]. It has been previously proposed that the existence of flavonoids in the leaf of *C. roseus* may consider its remarkably high radical scavenging activity [93,94]. The presences of these phytochemicals in plants (terpenoids, tannins, alkaloids) display an excellent relationship for the antimicrobial activities of the plants [95]. Moon et al. [96] demonstrated the anticancer activity of *C. roseus* against the human renal cell carcinoma Caki-2 cell line and a non-cancer MDCK cell line from Madin-Darby canine kidneys, and they attributed this effective anticancer activity to the presence of bioactive compounds and the terpenoid indole acetic acid. Drought and heat stress significantly reduced the antimicrobial and anticancer activities of plant extracts in the present study. This result could be attributed to the reduction in phenolics and flavonoids contents recorded under drought and heat stress.

## 4. Conclusions

Drought and heat stress significantly decreased growth and biomass accumulation in *Mentha piperita* and *Catharanthus roseus.* Increased accumulation of osmolytes and secondary metabolites at early and late growth stages in response to stress confirmed the potential of these compounds to counteract the deleterious effects of drought and heat stress. The accumulation of osmolytes and secondary metabolites may contribute to the maintenance of growth under stress conditions by improving the plant water potential and increasing ROS scavenging, thereby preventing the damaging effects of drought and heat stress. Aqueous and methanolic extracts of both plant species showed significant antibacterial, antifungal, and anticancer activity, confirming the promising medicinal potential of these two plants.

## Figures and Tables

**Table 1:** Effect of drought, heat and drought + heat stress on the height of *M. piperita* and *C. roseus* after seven and fourteen days of stress exposure.

Plant	Treatments	Height (cm)	
		7 Day	14 Day
*M. piperitas*	Control	54.8 ± 0.42 a	61.4 ± 0.61 a
	Drought	44.9 ± 0.10 bc	53.6 ± 0.26 b
	Heat	47.6 ± 0.19 b	55.4 ± 0.30 b
	Drought + Heat	36.6 ± 0.28 d	43.6 ± 0.02 c
*C. roseus*	Control	35.2 ± 0.27 d	43.8 ± 0.16 c
	Drought	29.4 ± 0.40 ef	34.3 ± 0.20 e
	Heat	31.7 ± 0.20 e	39.5 ± 0.11 d
	Drought + Heat	19.3 ± 0.21 g	22.5 ± 0.26 f

Mean (±SE) of five values is presented and values denoted by different letters are significantly different at *p* < 0.05.

**Table 2:** Effect of drought, heat and drought + heat stress on the fresh and dry weight of shoot and root of *M. piperita* and *C. roseus* after seven and fourteen days of stress exposure.

Plant	Treatments	Shoot FW (g)		Root FW (g)		Shoot DW (g)		Root DW (g)	
		7 Day	14 Day	7 Day	14 Day	7 Day	14 Day	7 Day	14 Day
*M. piperita*	Control	20.4 ± 0.13 a	26.6 ± 0.23 a	8.4 ± 0.032 a	9.1 ± 0.06 a	1.2 ± 0.026 a	1.7 ± 0.028 a	0.6 ± 0.013 a	0.7 ± 0.035 a
	Drought	12.4 ± 0.31 c	19.2 ± 0.84 b	6.3 ± 0.012 b	7.5 ± 0.02 c	0.9 ± 0.017 c	1.4 ± 0.126 b	0.5 ± 0.017 b	0.6 ± 0.024 c
	Heat	17.1 ± 0.24 b	15.3 ± 0.85 c	6.7 ± 0.14 b	8.2 ± 0.03 ab	1.1 ± 0.011 b	1.3 ± 0.026 b	0.4 ± 0.023 d	0.6 ± 0.021 b
	Drought + Heat	10.3 ± 0.14 d	14.7 ± 0.14 c	5.7 ± 0.063 c	6.4 ± 0.033 d	0.7 ± 0.022 e	0.9 ± 0.066 c	0.5 ± 0.022 bc	0.5 ± 0.023 d
*C. roseus*	Control	11.4 ± 0.61 cd	12.3 ± 0.18 d	4.1 ± 0.044 d	6.2 ± 0.02 d	1.1 ± 0.051 b	1.4 ± 0.44 b	0.5 ± 0.016 b	0.5 ± 0.012 d
	Drought	7.2 ± 0.25 ef	9.3 ± 0.13 f	3.6 ± 0.033 e	4.8 ± 0.01 e	0.7 ± 0.028 e	0.8 ± 0.026 d	0.3 ± 0.024 d	0.3 ± 0.024 f
	Heat	8.1 ± 0.61 e	10.6 ± 0.07 e	3.8 ± 0.065 e	5.1 ± 0.05 e	0.8 ± 0.028 d	0.8 ± 0.031 d	0.4 ± 0.023 d	0.4 ± 0.014 e
	Drought + Heat	5.1 ± 0.45 g	8.1 ± 0.06 fg	2.8 ± 0.082 f	3.2 ± 0.07 f	0.7 ± 0.017 e	0.8 ± 0.035 d	0.3 ± 0.017 f	0.3 ± 0.022 g

Mean (±SE) of five values is presented and values denoted by different letters are significantly different at *p* < 0.05. FW denotes fresh weight; DW denotes dry weight.

**Table 3:** Diameter of inhibition zone (mm) of *M. piperita* and *C. roseus* against three pathogenic bacterial isolates under control and 14 day stress conditions.

Bacterial Strain	Plants	Treatment	Control		Drought		Heat		Drought + Heat		0.2µg/mL Ampicillin
			10%	20%	10%	20%	10%	20%	10%	20%	
*P. aeruginosa*	*M. piperita*	Aqueous	13 ± 0.23 d	18 ± 0.27 b	11 ± 0.24 f	15 ± 0.21 c	10 ± 0.12 g	12 ± 0.11 e	12 ± 0.24 e	11 ± 0.24 f	32 ± 0.21 a
		MeOH	15 ± 0.17 e	20 ± 0.24 b	14 ± 0.21 f	18 ± 0.24 c	12 ± 0.17 g	17 ± 0.22 d	11 ± 0.21 h	17 ± 0.22 d	36 ± 0.22 a
	*C. roseus*	Aqueous	16 ± 0.25 e	27 ± 0.21 a	14 ± 0.22 f	24 ± 0.24 b	12 ± 0.21 g	21 ± 0.22 c	12 ± 0.21 g	22 ± 0.21 c	19 ± 0.24 d
		MeOH	14 ± 0.27 e	31 ± 0.22 b	12 ± 0.21 f	27 ± 0.21 c	9 ± 0.11 h	24 ± 0.26 d	10 ± 0.26 g	23 ± 0.22 d	37 ± 0.25 a
*S. aureus*	*M. piperita*	Aqueous	12 ± 0.31 f	19 ± 0.29 b	11 ± 0.25 g	16 ± 0.22 c	10 ± 0.21 g	14 ± 0.25 d	8 ± 0.22 h	13 ± 0.24 e	38 ± 0.22 a
		MeOH	16 ± 0.18 e	25 ± 0.27 b	13 ± 0.24 f	21 ± 0.23 c	12 ± 0.24 g	20 ± 0.21 c	10 ± 0.21 h	18 ± 0.21 d	30 ± 0.21 a
	*C. roseus*	Aqueous	23 ± 0.22 e	36 ± 0.26 a	20 ± 0.22 f	32 ± 0.25 b	17 ± 0.27 g	29 ± 0.22 c	15 ± 0.25 h	27 ± 0.22 d	27 ± 0.22 d
		MeOH	25 ± 0.27 f	37 ± 0.22 a	23 ± 0.27 g	34 ± 0.24 b	20 ± 0.21 h	30 ± 0.25 d	19 ± 0.22 i	31 ± 0.25 c	27 ± 0.21 e
*R. solanacearum*	*M. piperita*	Aqueous	18 ± 0.24 c	23 ± 0.18 a	15 ± 0.22 e	20 ± 0.26 b	14 ± 0.24 e	18 ± 0.24 c	14 ± 0.21 e	17 ± 0.22 d	24 ± 0.21 a
		MeOH	19 ± 0.26 d	26 ± 0.32 a	18 ± 0.22 e	24 ± 0.24 b	16 ± 0.26 f	21 ± 0.22 c	17 ± 0.22 f	22 ± 0.21 c	27 ± 0.25 a
	*C. roseus*	Aqueous	15 ± 0.21 f	25 ± 0.33 b	13 ± 0.21 g	23 ± 0.22 c	11 ± 0.25 h	20 ± 0.21 d	10 ± 0.21 i	19 ± 0.22 e	28 ± 0.14 a
		MeOH	22 ± 0.25 c	26 ± 0.31 b	20 ± 0.27 d	22 ± 0.22 c	17 ± 0.21 e	23 ± 0.22 c	15 ± 0.24 f	20 ± 0.24 d	28 ± 0.21 a

Mean (±SE) of five values is presented and values denoted by different letters are significantly different at *p* < 0.05.

**Table 4:** Effect of different concentrations of 0.5% and 1.0% (v:v) of *M. piperita* and *C. roseus* Aqueous and methanolic extracts on the growth of *F. oxysporum* and *A. terreus* under control and 14 day stress conditions. Mean diameters are expressed in mm.

Fungal Strain	Plants	Treatment	Control		Drought		Heat		Drought + Heat		Rhizolex-T
			0.5%	1.0%	0.5%	1.0%	0.5%	1.0%	0.5%	1.0%	
*F. oxysporum*	*M. piperita*	Aqueous	74 ± 0.92 e	72 ± 0.46 f	77 ± 0.76 c	75 ± 0.65 d	79 ± 0.87 b	78 ± 0.77 b	80 ± 0.43 a	78 ± 0.76 b	48.6 ± 0.55
		MeOH	58 ± 0.87 f	53 ± 0.92 g	64 ± 0.79 d	61 ± 0.85 e	67 ± 0.83 b	63 ± 0.81 d	69 ± 0.85 a	65 ± 0.91 c	
	*C. roseus*	Aqueous	52 ± 0.68 e	46 ± 0.64 f	56 ± 0.92 d	53 ± 0.86 e	61 ± 0.91 b	56 ± 0.66 d	63 ± 0.72 a	60 ± 0.86 c	
		MeOH	47 ± 0.54 e	43 ± 0.86 f	52 ± 0.75 d	47 ± 0.83 e	56 ± 0.85 c	50 ± 0.73 c	59 ± 0.91 a	57 ± 0.74 b	
*A. terreus*	*M. piperita*	Aqueous	63 ± 0.76 e	60 ± 0.93 f	68 ± 0.76 c	64 ± 0.76 d	70 ± 0.64 b	67 ± 0.83 c	74 ± 0.82 a	70 ± 0.76 b	26.6 ± 0.43
		MeOH	60 ± 0.73 d	56 ± 0.38 f	66 ± 0.67 c	59 ± 0.65 e	69 ± 0.97 b	67 ± 0.86 c	73 ± 0.59 a	69 ± 0.92 b	
	*C. roseus*	Aqueous	64 ± 0.48 e	53 ± 0.86 f	68 ± 0.92 d	64 ± 0.87 e	71 ± 0.65 b	69 ± 0.92 c	73 ± 0.23 a	71 ± 0.81 b	
		MeOH	60 ± 0.89 f	56 ± 0.74 g	67 ± 0.54 d	64 ± 0.76 e	69 ± 0.85 b	67 ± 0.76 d	71 ± 0.67 a	68 ± 0.73 c	

Mean (±SE) of five values is presented and values denoted by different letters are significantly different at *p* < 0.05.

**Table 5:** Percentage of inhibition (%) of PC3 and MCF-7 cancer cell lines exposed to different concentrations (50 and 100 µg/mL) of *M. piperita* and *C. roseus* aqueous and methanolic extracts under control and 14 day stress conditions.

Cell Lines	Plants	Treatment	Control		Drought		Heat		Drought + Heat	
			50 µg/mL	100 µg/mL	50 µg/mL	100 µg/mL	50 µg/mL	100 µg/mL	50 µg/mL	100 µg/mL
PC3	*M. piperita*	Aqueous	56 ± 0.76 b	64 ± 0.72 a	48 ± 0.71 e	53 ± 0.77 c	43 ± 0.43 f	50 ± 0.43 d	44 ± 0.88 f	48 ± 0.51 e
		MeOH	50 ± 0.83 b	65 ± 0.82 a	44 ± 0.62 d	47 ± 0.82 c	41 ± 0.75 e	43 ± 0.76 d	38 ± 0.54 f	41 ± 0.44 e
	*C. roseus*	Aqueous	46 ± 0.59 c	61 ± 0.81 a	42 ± 0.73 e	47 ± 0.77 b	39 ± 0.73 f	44 ± 0.83 d	37 ± 0.77 g	42 ± 0.54 e
		MeOH	38 ± 0.73 d	56 ± 0.73 a	35 ± 0.71 e	42 ± 0.82 b	33 ± 0.82 g	38 ± 0.84 d	34 ± 0.82 f	39 ± 0.76 c
MCF-7	*M. piperita*	Aqueous	63 ± 0.83 c	81 ± 0.62 a	57 ± 0.55 f	65 ± 0.73 b	53 ± 0.47 g	61 ± 0.81 d	51 ± 0.77 h	59 ± 0.82 e
		MeOH	53 ± 0.81 c	72 ± 0.81 a	50 ± 0.65 e	56 ± 0.76 b	47 ± 0.92 g	52 ± 0.82 d	49 ± 0.43 f	49 ± 0.57 f
	*C. roseus*	Aqueous	60 ± 0.73 d	74 ± 0.62 a	55 ± 0.82 e	70 ± 0.82 b	52 ± 0.82 f	64 ± 0.65 c	49 ± 0.76 g	60 ± 0.74 d
		MeOH	45 ± 0.61 e	67 ± 0.71 a	41 ± 0.56 f	62 ± 0.84 b	37 ± 0.65 g	57 ± 0.84 c	36 ± 0.45 h	54 ± 0.76 d

Mean (±SE) of five values is presented and values denoted by different letters are significantly different at *p* < 0.05.
